# Material Properties of Composite Resins Used for Orthodontic Attachments in Clear Aligner Therapy: A Systematic Review

**DOI:** 10.3390/biom16060822

**Published:** 2026-06-02

**Authors:** Lara Frias, Rita Fidalgo-Pereira, Rita Noites, Maria J. Correia, Ana T. P. C. Gomes, Pedro C. Lopes

**Affiliations:** 1 Faculty of Dental Medicine, Universidade Católica Portuguesa, Estrada da Circunvalação, 3504 505 Viseu, Portugal; larabastosfrias@gmail.com (L.F.); rfpereira@ucp.pt (R.F.-P.); rita.noites@ucp.pt (R.N.); mcorreia@ucp.pt (M.J.C.); apgomes@ucp.pt (A.T.P.C.G.); 2 Faculty of Dental Medicine, Center for Interdisciplinary Research in Health (CIIS), Universidade Católica Portuguesa, 3504 505 Viseu, Portugal

**Keywords:** clear aligners, orthodontic attachments, composite resins, biomaterials, mechanical properties, wear resistance

## Abstract

Clear aligner therapy has become increasingly widespread in contemporary orthodontics, relying on composite resin attachments to enhance force transmission and improve the predictability of tooth movement. The physicochemical and mechanical properties of these biomaterials play a crucial role in attachment durability, dimensional stability, and esthetic performance during treatment. This systematic review aimed to evaluate how different composite resin types influence the mechanical, optical, and functional performances of orthodontic attachments used in clear aligner therapy. A systematic literature search was conducted in the PubMed, Scopus, and Cochrane databases for studies published between 2015 and 2025, following PRISMA guidelines. In vitro studies and clinical trials evaluating composite resins used for attachment fabrication were included. Fifteen studies met the eligibility criteria, including eleven laboratory investigations and four clinical studies. The evaluated outcomes comprised shear bond strength, wear resistance, surface roughness, microhardness, color stability, and accuracy of attachment reproduction. Overall, all evaluated composite resins demonstrated shear bond strength values within clinically acceptable ranges. However, significant differences were observed in the material performances depending on the resin composition and viscosity. Nanohybrid and high-viscosity composite resins were generally associated with improved mechanical resistance, reduced wear, and greater dimensional stability, although SBS outcomes should be interpreted in light of the bonding protocols used. In contrast, flowable composite resins showed improved handling and adaptation to attachment molds but presented higher susceptibility to surface degradation and discoloration. The findings suggest that the composition and properties of composite resins significantly influence the mechanical and optical behavior of orthodontic attachments. Optimizing material selection according to biomechanical demands and esthetic requirements may improve attachment longevity and treatment predictability in clear aligner therapy. Clinicians should prioritize nanohybrid or high-viscosity composite resins for high-load attachments and use flowable composite resins materials when adaptation and esthetics are critical.

## 1. Introduction

Orthodontic treatment aims to correct dental malocclusions while restoring functional occlusion and improving facial esthetics [1]. In recent years, clear aligner therapy has gained widespread acceptance as a discreet and patient-friendly orthodontic approach. This treatment modality integrates digital technologies, including intraoral scanning, computer-aided design (CAD), and thermoforming processes, to produce individualized orthodontic appliances with high precision and esthetic appeal [2,3,4].

The materials used in clear aligner systems are typically polyurethane and glycol-modified polyethylene terephthalate (PET-G), due to their mechanical strength, elastic behavior, and favorable biocompatibility profile [4,5]. These polymers maintain dimensional stability and optical clarity throughout treatment [6,7]. Additionally, the hydrophobic characteristic of PET-G reduces bacterial adhesion, decreasing the risk of biofilm formation on the aligner surface [8,9]. In many cases, these materials are modified or combined to improve aligner performance. Some manufacturers develop multilayer aligners, which combine more flexible inner layers with more rigid outer layers, balancing comfort and the effective application of orthodontic forces [9].

When selecting orthodontic treatment modalities, biological- and material-related factors must also be considered. Clear aligner therapy differs from conventional fixed appliances in terms of oral hygiene maintenance, since aligners can be removed during meals and brushing, reducing biofilm accumulation and facilitating periodontal health. This characteristic contributes to the growing popularity of aligner-based orthodontic treatment [10]. In addition to aligners, this system is also composed of attachments that are composite resin elements applied to the buccal surfaces of teeth during orthodontic treatment with aligners. They act as support and retention points, allowing for more controlled and complex tooth movements, improving the efficiency of force transmission. In the context of treatment planning, the morphological characteristics, dimensional parameters, and constituent materials of the attachments must be rigorously assessed to ensure predictable outcomes, optimal stability, and overall clinical success [11,12]. Orthodontic attachments play a fundamental role in clear aligner therapy by acting as biomechanical interfaces that enhance the transmission of controlled and programmed forces from the aligner to the tooth surface [11,13]. These composite resin structures are indicated to facilitate complex tooth movements, such as rotations, extrusions, and torque control, which cannot be efficiently achieved with aligners alone [11,12].

Clinically, attachments are fabricated using a direct bonding technique, in which a composite resin is inserted into a prefabricated template—typically made of thermoformed polyethylene terephthalate glycol (PET-G)—and positioned over the teeth before light polymerization [8,13]. The accuracy of this process depends not only on the geometric design of the attachment but also on the physicochemical properties of the composite resin, including the viscosity, filler content, and polymerization behavior [14,15].

Therefore, the effectiveness of the attachment-based biomechanics is determined not only by the digital treatment planning but also by the material’s ability to accurately reproduce the planned shape, resist wear, and maintain dimensional stability under continuous mechanical interaction with the aligner [13,16]. Orthodontic attachments are typically fabricated using different types of composite resins, including flowable, conventional nanohybrid materials. Each type presents distinct mechanical and handling properties that may influence attachment performance. In clinical practice, complications such as attachment fracture, debonding, wear, or loss of shape may occur during treatment, potentially compromising force transmission and reducing the predictability of tooth movement. These issues highlight the importance of selecting appropriate materials for attachment fabrication and justify the need for further investigation regarding the influence of the composite resin type on aligner treatment outcomes [11,13,17].

Attachments are available in different types, shapes, and functions, playing an essential role in the transmission of specific and controlled dental forces. For these movements to be executed accurately, it is imperative that attachments are fabricated using composite resins with high durability and mechanical performance. The careful selection of the appropriate material for each type of attachment, considering its position and clinical function, is crucial for efficacy, allowing for a reduction in the total treatment duration for the patient and optimizing the clinical intervention time. Correct adaptation of the composite resin to the dental morphology, combined with standardized application techniques, is critical to minimize failures and optimize aligner performance. Other relevant factors include the chemical and thermal resistance of the material in the oral environment, biocompatibility, the capacity for complete polymerization, and dimensional stability over time, all of which directly impact the longevity of the attachment and the maintenance of applied forces [13,17].

This systematic review aimed to evaluate whether different types of composite resins used for attachment fabrication influence the mechanical, esthetic, and clinical performance of clear aligner therapy. The main outcomes analyzed were accuracy of shape reproduction and fit, shear bond strength, wear and volumetric loss over time, and color stability, as well as their overall impact on treatment efficiency and duration.

## 2. Materials and Methods

### 2.1. Search Strategy

A comprehensive and systematic literature search was conducted in September 2025 on the electronic databases PubMed, Scopus, and Cochrane to identify relevant studies on the use of different types of composite resins in the fabrication of attachments. This systematic review was carried out in accordance with the PRISMA (Preferred Reporting Items for Systematic Reviews and Meta-Analyses) guidelines. Previous systematic reviews and the review protocol are registered in the OSF database under the Registration DOI https://doi.org/10.17605/OSF.IO/EHKRB (accessed on 8 October 2025).

The research question was formulated according to the PICO strategy, defined as follows:

P (Population): Patients undergoing orthodontic treatment with transparent aligners.

I (Intervention): Use of different types of composite resins in the fabrication of attachments.

C (Comparison): Distinct composite resin materials.

O (Outcome): Impact on the efficacy of clear aligners, considering parameters such as:

- Accuracy of shape and fit reproduction;
- Shear strength;
- Wear over time;
- Durability and dimensional stability;
- Color stability;
- Influence on treatment efficiency and duration.

Below is the defined PICO question:

In patients undergoing orthodontic treatment with clear aligners, does the use of different types of composite resins for attachment fabrication, compared with other composite materials, influence the effectiveness of orthodontic treatment?

The search strategy was initially constructed for the PubMed database and subsequently adapted for each of the other databases consulted. The results obtained were cross-checked to identify and remove duplicates. The search equation used was as follows: (orthodontic attachment OR attachment) AND (composite resins OR composite) AND (clear aligner OR aligner OR Invisalign).

Period: articles published within the last 10 years (2015–2025);

Search date: 14 September 2025.

In total, the search identified 215 articles in PubMed, 54 in Scopus, and 30 in Cochrane.

Inclusion criteria:

- Clinical trials.
- In vitro studies.
- Published between 2015 and 2025.
- Studies addressing the comparison of different types of composite resins in the fabrication of attachments.
- Full-text availability.

Exclusion criteria:

- Systematic reviews.
- Critical or narrative reviews.
- Letters to the editor, editorials, or clinical guidelines.

### 2.2. Study Selection and Data Extraction

The study selection process was performed in three stages:

- Initial screening: Two independent reviewers (P.L. and L.F.) examined the titles and abstracts of the identified articles, applying the predefined eligibility criteria. Any discrepancies between the reviewers were resolved through discussion with a third author (R.F.-P.). Cohen’s Kappa test was performed to assess the inter-reviewer agreement. The Rayyan Intelligent Systematic Review Platform was used to assist in the systematic review process [18].
- Abstract reading: Non-excluded articles were evaluated based on their abstracts to confirm compliance with the inclusion criteria before full-text assessment.
- Full evaluation: Potentially eligible articles were read in full and analyzed for their relevance to the review objective.

Included studies were assigned specific codes for organization and analysis.

During data extraction, the following information was collected and organized in a table: article title, year of publication, authors, methodological design, sample characteristics, type of orthodontic treatment, duration of clinical follow-up, and evaluated outcomes.

### 2.3. Methodological Quality Assessment

The methodological quality of the included studies was evaluated using the Revised Cochrane Risk-of-Bias Tool for Randomized Trials (RoB 2.0).This tool comprises five main domains:

D1: bias arising from the randomization process.

D2: bias due to deviations from the intended interventions.

D3: bias due to missing outcome data.

D4: bias in the measurement of outcomes.

D5: bias in the selection of the reported results.

Each study was individually assessed with respect to these domains, and one of the following judgments was assigned: low risk of bias, some concerns, or high risk of bias.

The final classification of each study was determined based on the predominant judgment across the five domains, thereby reflecting the overall risk of bias assigned.

## 3. Results

### 3.1. Study Selection

The initial search identified a total of 299 articles, of which 215 were retrieved from PubMed, 54 from Scopus, and 30 from the Cochrane Library. After the removal of 49 duplicates, 250 articles remained for screening.

In the first evaluation stage, based on titles and abstracts, 233 articles were excluded for the following reasons: (1) systematic review, (2) animal study, (3) wrong study design, (4) wrong outcomes and (5) article not related to the theme.

**Figure 1:**
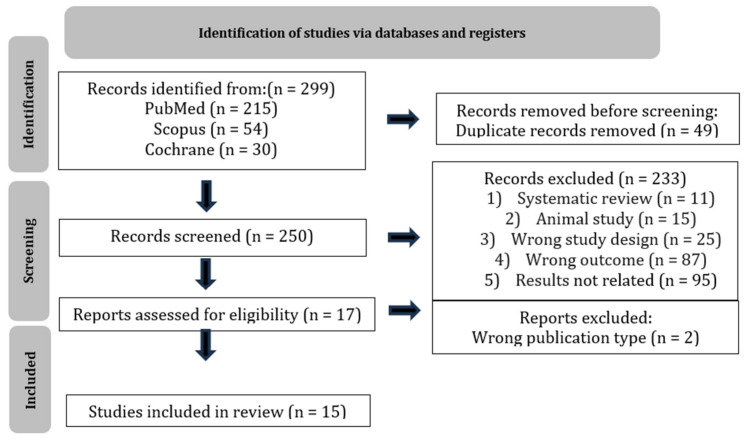
Study selection process illustrated in PRISMA flow diagram.

Thus, 17 articles were selected for full-text reading. Of these, two studies were subsequently excluded because they were only study registrations that had not yet been completed and had no published results. At the end of the selection process, 15 studies met all the eligibility criteria and were included in this review. Figure 1 shows the selection process according to the PRISMA guidelines, and Table 1 and Table 2 briefly present the main characteristics of the studies included in this systematic review.

None of the included clinical studies clearly reported sample sizes or follow-up durations. This lack of essential methodological information limits the ability to directly compare results across studies and reduces the overall strength and reliability of the clinical evidence.

A total of 15 articles published between 2019 and 2025 were included, analyzing different composite resins used in the fabrication of attachments for clear aligners. Of this total, 11 corresponded to in vitro studies and four to clinical trials conducted on patients. The included clinical studies were limited in number (*n* = 4) and presented variability in their follow-up durations, sample sizes, and evaluation criteria, which affects the comparability of the outcomes and the strength of the clinical conclusions.

In vitro studies mainly focused on the shear bond strength (SBS), surface wear, color stability, surface roughness, microhardness, and reproduction accuracy of the attachments. To facilitate a structured comparison across studies, the evaluated outcomes were categorized and are summarized in Table 3 according to their mechanical, optical, and functional relevance. The clinical trials primarily evaluated color change in the oral cavity, wear over time, and esthetic perception by patients. Among the composite resins studied, Filtek™ Z350 XT (3M ESPE) was the most frequently evaluated, in its different formulations (flowable, universal, nanoparticulate composite resins). Other recurrent materials included GC Aligner Connect, Tetric range composites (Ivoclar/GC) in different viscosities, Amelogen Plus (Ultradent), Omnichroma (Tokuyama), and G-ænial Universal Injectable (GC). Overall, all studies reported adhesive strength values considered clinically acceptable for use in attachments. Most investigations showed that flowable composite resins offer greater ease and speed of handling, while high-viscosity composite resins (nanohybrid) demonstrated greater wear resistance and smoother surfaces.

Regarding color stability, significant changes were observed after exposure to staining agents, with coffee and red wine causing the most discoloration.

Concerning the accuracy of attachment reproduction, laboratory studies showed differences between materials and polymerization techniques, with variation in the presence of excess composite resin and in the fidelity of digitally planned shapes.

### 3.2. Risk-of-Bias Analysis

Risk-of-bias analysis was carried out for all included studies, with the purpose of assessing the methodological quality and the reliability of the findings.

For clinical trials, the Cochrane RoB 2 tool was used, while for in vitro studies, an adaptation of the criteria proposed by [31] was applied, which consider aspects such as randomization, blinding, standardization of the experimental protocol, and adequacy of statistical analysis (Table 4).

Most of the studies included presented a low risk of bias, reflecting consistent methodologies, well-defined criteria, and appropriate outcome measurements. Studies such as those by [20,21,23,27,28] were classified as having low risk in all domains, demonstrating methodological robustness and transparency in experimental conduct.

Some studies, such as [19,26,29,30], presented “some concerns”, mainly due to the absence of detailed information on the randomization process, the possible influence of deviations from the original intervention, or outcome measurement without blinding. Nevertheless, the potential biases identified do not substantially compromise the validity of the presented results. Overall, the evaluated studies show good methodological quality, with low overall risk of bias, supporting the reliability of the conclusions of this systematic review.

## 4. Discussion

The results of this review demonstrate that the mechanical properties of composite resins influence the durability of orthodontic attachments. In the specific context of orthodontic attachments, the filler size and shape, monomer composition, resin matrix viscosity and polymerization conditions should not be regarded as generic material descriptors. Rather, these parameters directly influence the attachment shape fidelity, aligner seating, wear resistance during repeated aligner insertion and removal, and stability of force transmission throughout treatment [11,12,13]. A structured comparison of the evaluated outcomes across studies is presented in Table 3, allowing for direct visualization of the compositions of different composite resins and the mechanical and optical properties.

Regarding SBS, most studies maintained identical adhesive systems and polymerization conditions across the tested composite resins, allowing for intra-study comparison of the material performance. For example, ref. [19] standardized both the adhesive protocol and curing conditions, reporting SBS values between 10 and 20 MPa, with Filtek™ P60 exhibiting the highest values. Similarly, ref. [25] applied a consistent adhesive strategy and light-curing protocol, with Tetric N-Ceram showing the highest SBS values (21 ± 4 MPa). Nevertheless, substantial variability in adhesive systems and bonding protocols was observed between studies. Therefore, although higher SBS values were frequently associated with nanohybrid and high-viscosity composite resins, these findings should be interpreted cautiously, since bond strength may also be influenced by the adhesive strategy, enamel conditioning, curing parameters, and testing methodology. The higher SBS values may be partly associated with increased inorganic filler contents, reduced organic matrix proportions, and lower polymerization shrinkage stress, resulting in the stronger stability of orthodontic attachments. Indeed, Filtek™ P60 (3M) is composed of 83 wt% inorganic filler particles of zirconia and silica in the range of 0.01–3.5 μm, and Tetric N-Ceram (Ivoclar) has a filler load of approximately 81 wt%. Also, Tetric N-Ceram (Ivoclar) and Filtek™ P60 (3M) share the same monomers within the organic matrix UDMA, BIS-EMA and Bis-GMA, which can also increase the inherent rigidity and decrease polymerization shrinkage [17,18,19]. Nano- and micro-scale particles are combined in the composite resins’ microstructure to promote mechanical reinforcement under chewing loading [14,16,19]. SBS value differences may be related to the filler morphology, filler loading, resin matrix viscosity, or degree of conversion, which may enhance substrate adaptation and stress distribution during polymerization, potentially influencing the SBS at the interface. However, the interpretation of bond strength outcomes should be approached cautiously, since this parameter may also be influenced by adhesive system, polymerization parameter, substrate-conditioning and adhesive protocol variability among studies. In contrast, flowable composite resins, such as Filtek Z350 XT Flow and GC Aligner Connect, showed greater ease of handling and adaptation to the molds but presented lower mechanical resistance and higher surface roughness [14,19,20]. This difference can be explained by the higher amount of triethylene glycol dimethacrylate (TEGDMA) and bisphenol A ethoxylated dimethacrylate (Bis-EMA) within the organic matrix, responsible for reducing viscosity and increasing flowability. In orthodontic attachments, this balance becomes clinically critical, as excessive flowability may lead to distortion or excess material during template placement, compromising attachment shape accuracy and aligner fit. Conversely, higher-viscosity materials contribute to better shape retention and more predictable force application throughout treatment [14,15].

The dimensional reliability of attachments depends on multiple factors, including the viscosity of the composite resin, the mold material, and the photopolymerization conditions [14,20]. The study by [21] demonstrated that PET-G molds presented greater dimensional stability and precision in reproducing shapes compared to polyethylene molds. This result is consistent with the physical properties of PET-G, which features high rigidity and lower thermal deformation [8,20].

Regarding viscosity, it was observed that the Filtek Z350 Universal composite resin with 72.5 wt% of inorganic filler particles exhibited higher shape fidelity with less overflow, while the flowable composite resin Z350 XT, with 65 wt% of inorganic particles, showed a tendency toward excess volume formation, especially under a reduced irradiance time of 10 s [20]. Found that Filtek Z350 XT Universal (3M) presented smoother surfaces and lower roughness, while GC Aligner Connect™ showed greater roughness under high irradiance of 3200 mw/cm^2^ for 3 s [20]. The time of light exposure depends on the light irradiance as well as on the type and thickness of the restorative materials to reach the energy required for the polymerization of composite resins. Higher irradiance energy not only increases the degree of conversion and surface hardness but may also induce thermal stresses and microcracks [21,22]. Previous studies have shown that the adequate polymerization of composite resins depends on both the irradiance of the light-curing unit and the exposure time. An irradiance close to 1000 mW/cm^2^, combined with sufficient curing time, is generally recommended to achieve appropriate radiant exposure and clinically accepted degree-of-conversion values between 52 and 75% immediately post-polymerization [21,22]. In the context of attachment fabrication, inadequate polymerization may result not only in reduced mechanical performance but also in subtle dimensional inaccuracies, which can compromise the aligner seating and the efficiency of the force transmission [21,22]. Thus, standardizing the curing protocol with adequate light irradiation and exposure time, visible light irradiance and wavelengths, and the correct distance between LCU tips are essential to avoid variations in attachment accuracy.

The color stability of composite resins is a fundamental parameter in material selection, especially for esthetic areas. In general, studies indicate that coffee and red wine are the most aggressive staining agents, inducing color changes beyond clinically perceptible thresholds [17,20,23]. In clinical trials, ref. [28] confirmed that G-ænial Universal Injectable showed less color change after exposure to staining substances, a result attributed to its high inorganic filler density, filler distribution, particle shape and size, and low content of aromatic monomers. These findings reinforce that chemical composition and surface finishing are determining factors for esthetic performance, with the use of nanoparticulate composites being recommended in anterior regions [20,23]. The study by Erçin, ref. [25] demonstrated that Omnichroma (Tokuyama) showed the best performance in terms of color stability, followed by GC Aligner Connect, while Tetric PowerFlow exhibited the highest color change after immersion in coffee. These results corroborate previous findings by Feinberg et al. [24], who identified the direct influence of the matrix composition and the amount of nanometric particles on stain resistance.

Wear resistance is another crucial factor for the longevity of attachments. Continuous friction between the composite resin and the aligner can compromise the shape and force transmission. Ref. [23] observed that G-ænial Universal Flo exhibited the lowest volumetric wear, while Flow Tain showed the greatest volume loss. These results were corroborated by [14], who found lower wear and higher SBS for SonicFill compared to the Z350XT and Z350XT Flow composites [14,19]. Clinically, progressive wear of attachments may lead to loss of their designed morphology, reducing the effectiveness of the aligner engagement and ultimately compromising the predictability of tooth movement [13,16]. The superior wear resistance of hybrid and nanoparticulate composite resins is mainly attributed to their higher filler contents and more homogeneous distribution of smaller filler particles, which enhance resistance to wear and long-term stability [24,25,26]. Meanwhile, composite resins with a high proportion of organic matrix are more susceptible to hydrolytic degradation and microabrasion [21,25]. Thus, materials such as Filtek P60 and Tetric EvoCeram demonstrated more stable performances, especially in posterior attachments subjected to higher loads [14,16,19].

The adhesive strength values observed in the included studies ranged from 6 to 14 MPa, values considered clinically acceptable for use in orthodontic attachments [14,19]. Ref. [25] reported that all tested composite resins exceeded the recommended minimum value, with Tetric N-Ceram showing the highest SBS and Aligner Connect the lowest. Adhesive and cohesive failures were frequently reported, being related to tooth morphology, type of resin, and masticatory habits [26,27,28]. Ref. [29] observed that conventional attachments had a higher incidence of adhesive failures, while optimized attachments showed a higher prevalence of cohesive failures, reflecting the internal structural behavior of the material. This is directly related to attachment loss, which is more common in posterior teeth and can be minimized through proper isolation during the application and selection of composite resins with higher elastic moduli [13,14,16].

Although this review focused on the intrinsic properties of composite resins used for orthodontic attachment fabrication, adhesion to enamel is not determined by the composite resin alone [29]. Bonding agents represent the true adhesive interface between enamel and the composite attachment and may substantially influence the shear bond strength, debonding behavior, and clinical risk of attachment loss [30]. Therefore, differences in adhesive systems, enamel conditioning protocols, curing procedures, and isolation conditions across studies may act as confounding factors when comparing the performances of different composite resins. For this reason, the SBS results reported in this review should be interpreted as reflecting the performance of the complete bonding protocol rather than the composite resin in isolation. Future studies should standardize, or at least comprehensively report, the adhesive system, etching strategy, primer/adhesive application, light-curing protocol, and debonding assessment to allow for more reliable comparisons between composite materials.

The surface integrity of composite resins has a direct impact on biocompatibility and oral hygiene. Rough surfaces promote bacterial retention and staining, while well-polished composite resins exhibit lower microbial adhesion [10]. Despite the potential release of residual monomers, such as Bis-GMA and TEGDMA, there is no clinical evidence of significant toxicity associated with the composite resins used in attachments [19,31]. Attachments play an essential role in the three-dimensional control of tooth movements [11,12]. The choice of composite resin must consider the biomechanical function and anatomical region, prioritizing high-strength materials for posterior areas and greater color stability for anterior regions [11]. Additionally, proper hygiene instruction and regular monitoring by the orthodontist are essential to reduce biofilm accumulation and the risk of adhesive or inflammatory failures [29].

The comparative analysis between the results obtained in in vitro and in vivo studies demonstrates a general agreement in the observed trends, despite differences in the magnitudes of the effects. In the in vitro tests, it was found that conventional nanohybrid composite resins showed higher shear bond strength values, lower wear, and better shape fidelity [14,17,19]. The same was observed in most in vivo studies, in which these materials demonstrated greater dimensional stability and a lower incidence of adhesive failures during clinical use [16,28]. While bonding agents are essential to adhesion, this systematic review investigated associations between intrinsic composite resin properties and attachment performance rather than isolating the independent effect of each material variable. Reliable inter-study comparability requires the careful standardization of adhesion protocols and testing methodologies; otherwise, variability in shear bond strengths and debonding behaviors remains confounded. Such standardization ensures that observed differences are attributable to the composites’ physicochemical characteristics—namely, the monomer composition, filler architecture, and rheological properties—rather than to heterogeneity in the adhesive systems employed.

A significant limitation across the included clinical studies was the absence of clearly defined sample sizes and follow-up periods. These factors are critical for interpreting the clinical relevance and durability of the reported outcomes. The lack of standardized reporting hampers direct comparison between studies and weakens the overall level of evidence. Future research should prioritize methodological consistency, including well-defined sample sizes and longitudinal follow-up, to allow for more reliable and clinically meaningful comparisons. Moreover, because adhesive systems and bonding protocols varied substantially across the included studies, the independent contribution of the composite resin composition to SBS outcomes cannot be fully isolated.

Furthermore, while laboratory tests revealed significant differences in the microhardness and surface roughness between flowable and conventional composite resins [15,19,20], clinical trials indicated that these discrepancies tend to diminish in the oral environment [10]. The color stability observed in vitro was corroborated in vivo, although the degree of discoloration was slightly higher in the clinical context [17,20,23]. This emphasizes the high experimental heterogeneity among the studies included in this review.

Laboratory studies are essential tools for predicting the intrinsic behavior of materials but do not replace in vivo evaluation, where interaction with the biological environment, functional wear, and individual patient variations can significantly modify the clinical performance of the attachments [13,29].

The main limitation of this review lies in the heterogeneity of methodologies specifically related to attachment fabrication, including differences in mold materials, photopolymerization protocols, and testing methods for wear and dimensional accuracy [14,19,23]. Additionally, most clinical studies evaluate attachment performance only during the early stages of treatment, which prevents assessment of long-term degradation under continuous aligner-induced friction [13]. This limitation is particularly relevant for orthodontic attachments, as their functional performance depends on maintaining shape integrity throughout the entire treatment duration. In general, nanohybrid and high-viscosity composite resins demonstrate better mechanical performances and lower wear, while flowable composite resins offer greater ease of handling and precision of adaptation. Customization of the material according to clinical needs, combined with standardized photopolymerization protocols and controlled application techniques, is essential to maximize the durability, effectiveness, and predictability of orthodontic treatments with clear aligners.

From a clinical perspective, understanding the mechanical and esthetic performances of different composite resins is essential for optimizing attachment longevity and improving treatment predictability in clear aligner therapy.

Regarding long-term performance, most available clinical studies present relatively short follow-up periods, which limits definitive conclusions about attachment longevity [13]. Nevertheless, based on the analyzed evidence, composite resins with higher organic matrix contents, such as flowable composite resins, are more susceptible to long-term wear and degradation due to their increased proportions of diluent monomers [25]. Therefore, in prolonged orthodontic treatments, the selection of materials with higher inorganic filler contents, such as nanohybrid composite resins, is critical to maintain attachment shape fidelity and ensure effective force transmission throughout treatment [24,25,26].

From a clinical translation perspective, the findings of this review support structured recommendations for practitioners. In areas subjected to higher masticatory loads (posterior teeth), nanohybrid or high-viscosity composite resins should be prioritized due to their superior wear resistance and dimensional stability [14,16,19]. In contrast, in esthetic zones (anterior teeth), materials with high filler contents and lower aromatic monomer contents, such as the G-ænial® Universal Injectable composite resin, should be preferred due to their improved color stability when exposed to staining agents such as coffee and red wine [20,23]. Additionally, standardization of the photopolymerization protocol, particularly using an irradiance close to 1000 mW/cm^2^ for 20 s, represents a critical clinical step to ensure that laboratory-derived mechanical properties are effectively translated into clinical success and reduced incidence of adhesive failures [21,22].

## 5. Conclusions

This systematic review evaluated the influence of the composite resin type on the performance of orthodontic attachments used in clear aligner therapy. The available evidence indicates that the physicochemical and mechanical properties of composite resins play a significant role in determining attachment stability, durability, and functional performance. Overall, nanohybrid and high-viscosity composite resins demonstrated superior mechanical properties, including higher shear bond strengths, improved wear resistance, and greater dimensional stability. In contrast, flowable composite resins showed advantages in handling and adaptation to attachment molds but were generally associated with increased surface degradation and discoloration over time. The findings highlight the importance of material composition, filler content, and polymerization protocols in optimizing the mechanical and optical behavior of orthodontic attachments. From a clinical perspective, the appropriate selection of composite resin according to biomechanical demands and esthetic requirements may contribute to improved treatment predictability and attachment longevity. Nevertheless, the current evidence is limited by methodological heterogeneity and the relatively small number of clinical studies. Future well-designed clinical trials with standardized evaluation protocols are needed to further clarify the long-term performance of composite resins used for orthodontic attachments.

## Figures and Tables

**Table 1:** The characteristics of the in vitro studies included in this systematic review.

Author (Year)	Composite Resins (Chemical Composition)	Fillers	Evaluated Parameters	Conclusions
[14]	Z350XT, 3M: Bis-GMA, UDMA, Bis-EMA Z350 Flow, 3M: Bis-GMA, UDMA, TEGDMA, Bis-EMA SonicFill, Kerr: Bis-GMA, Bis-EMA, EBPADMA, TEGDMA	Z350XT, 3M: silica, zirconia nanoparticles (20 μm) (72.5 wt%) Z350 Flow, 3M: ytterbium trifluoride, silica, zirconium oxide (65 wt%) SonicFill, Kerr: silicon dioxide-modified dimethacrylate aminoformate (81.3 wt%)	SBS, wear	SonicFill: best
[16]	Aligner Connect, GC: Octahydro-4,7-methano-1H-(indenediyl) bis(methylene) bismethacrylate, 1,3,5-triazine-2,4,6-triamine, polymer with formaldehyde, 2,2′-ethylene- dioxydiethyl dimethacrylate, 2-(H-benzotriazol-2-yl)-p-cresol, UDMA Ortho Connect: N.D. Z350 Flowable. 3M: Bis-GMA, TEGDMA, procrylat resin Z350 XT Universal, 3M: Bis-GMA, UDMA, Bis-EMA	GC Aligner Connect: N.D. Z350 Flowable: ytterbium trifluoride, silica, zirconium oxide (65 wt%) Z350 XT Universal, 3M: silica, zirconia nanoparticles (20 μm) (72.5 wt%) Ortho Connect: N.D.	Accuracy, roughness	Z350: smoother
[19]	Z250 XT, 3M: Bis-GMA, BIS-EMA, UDMA, TEGDMA Z350 XT, 3M: Bis-GMA, BIS-EMA, UDMA, PEGDMA, TEGDMA P60: Bis-GMA, UDMA, Bis-EMA	Z250 XT, 3M: 0.01–3.5 μm zirconia/silica (82 wt%) Z350 XT, 3M: 5–20 nm silica nanofillers and 0.6–1.4 μm zirconia/silica nanoclusters (78.5 wt%) P60: 0.01–3.5 μm zirconia/silica (83 wt%)	Color stability, SBS, wear	P60: best balance
[20]	GC Aligner, GC: Octahydro-4,7-methano-1H-(indenediyl) bis(methylene) bismethacrylate, 1,3,5-triazine-2,4,6-triamine, polymer with formaldehyde, 2,2′-ethylene- dioxydiethyl dimethacrylate, 2-(H-benzotriazol-2-yl)-p-cresol, UDMA Ortho Connect: N.D. Z350XT Flowable, 3M: Bis-GMA, TEGDMA, procrylat resin Z350XT Universal, 3M: Bis-GMA, UDMA, Bis-EMA	GC Aligner, GC: N.D. Ortho Connect: N.D. Z350XT Flowable, 3M: ytterbium trifluoride, silica, zirconium oxide (65 wt%) Z350XT Universal: silica, zirconia nanoparticles (20 μm) (72.5 wt%)	Adhesion, SBS	Z350 Universal: best SBS
[21]	Enaflow, Micerium: UDMA, HEMA Enamel Plus HRI, Micerium: tetramethylene dimethacrylate	Enaflow, Micerium: silicon dioxide Enamel Plus HRI, Micerium: 0.7–0.04 μm glass filler, silicone dioxide (77 wt%)	Accuracy	High viscosity, more stable
[22]	Tetric PowerFlow, Ivoclar: Bis-GMA, Bis-EMA, UDMA, DCP Tetric PowerFill, Ivoclar: Bis-GMA; Bis-EMA, UDMA, PBPA, DCP, β-allyl sulfone Filtek™ Supreme Flowable Restorative, 3M: procrylat, BisGMA, and TEG- DMA resins Filtek™ Supreme XTE Universal, 3M: Bis-GMA (5–10 wt%), UDMA, TEGDMA, Bis-EMA6, PEGDMA Clearfil Majesty Flow, Kuraray: TEGDMA, hydrophobic aromatic dimeth- acrylate dl-Camphorquinone · accelera- tors · pigments · others Estelite Sigma Quick, Tokuyama: Bis-GMA, TEGDMA	Tetric PowerFlow, Ivoclar: barium aluminum silicate glass, iso-filler copolymer mix, ytterbium fluoride (71 wt %) Tetric PowerFill, Ivoclar: barium aluminum silicate glass, iso-filler copolymer mix, ytterbium fluoride and spherical mixed oxides (79 wt%) Filtek™ Supreme Flowable Restorative, 3M: Non-agglomerated/non-aggregated surface-modified 20 nm silica filler; non-agglomer- ated/non-aggregated surface -modified 75 nm silica filler; surface-modified aggregated zirconia/silica cluster filler (comprising 20 nm silica and 4 to 11 nm zirconia particles); and ytterbium trifluoride filler with a range of particle sizes from 0.1 to 5.0 μm. The aggregate has an average cluster particle size of 0.6 to 10 μm (78.5 wt%). Clearfil Majesty Flow, Kuraray: silanated barium glass filler (average: 3 µm), silanated colloidal silica (average: 20 nm) (81 wt%) Estelite Sigma Quick, Tokuyama: SiO_2_, ZrO_2_, PFSC (200 nm) (82 wt%)	Microhardness, SBS	Clearfil Majesty Flow, Kuraray: best hardness
[23]	Flow Tain, Reliance: Bis-GMA, TEGDMA Transbond XT Light Cure Adhesive, 3M: Bis-GMA, TEGDMA G-aenial Universal Flo, GC: DMA, Bis-MPEPP Filtek Z350 XT Flowable Restorative, 3M: Bis-GMA, UDMA, TEGDMA, Bis-EMA	Flow Tain, Reliance: silica, barium glass, fumed silica (60 wt%) Transbond XT Light Cure Adhesive, 3M: silica, quartz (70 wt%) G-aenial Universal Flo: silica, strontium glass, fluoroaluminosilicate glass (69 wt%) Filtek Z350 XT Flowable Restorative, 3M: zirconia/silica (65 wt%)	Wear	G-aenial Universal Flo: best
[24]	Tetric N-Flow, Ivoclar: Bis-GMA, Bis-EMA, UDMA, Bis-PMA, DCP, D3MA Beautifil, Shofu: Bis-GMA, TEGDMA Z350XT, 3M: Bis-GMA, UDMA, Bis-EMA	Tetric N-Flow, Ivoclar: barium glass, ytterbium, trifuoride, copolymer, mixed oxides (SiO_2_/ZrO_2_) (79 wt%) Beautifil, Shofu: multifunctional glass filler, surface prereacted glass-ionomer filler based on aluminofluoro-borosilicate glass (83.3 wt%) Z350XT, 3M: silica, zirconia nanoparticles (20 μm) (72.5 wt%)	Wear	Z350: highest wear
[25]	Tetric Evoceram, Ivoclar: dimethacrylates Tetric N-Ceram, Ivoclar: dimethacrylates Tetric N-Flow, Ivoclar: Bis-GMA, Bis-EMA, UDMA, Bis-PMA, DCP, D3MA G-aenial Universal Injectable, GC: Bis-EMA, UDMA Aligner Connect, GC: Octahydro-4,7-methano-1H-(indenediyl) bis (methylene) bismethacrylate, 1,3,5-triazine-2,4,6- triamine, polymer with formaldehyde, 2,2′- ethylenedioxydiethyl dimethacrylate, 2-(2H- benzotriazol-2-yl)-p-cresol, UDMA… (not all content is shared)	Tetric Evoceram, Ivoclar: barium glass, ytterbium trifluoride, mixed oxides and copolymers (82 wt%) Tetric N-Ceram, Ivoclar: barium glass, ytterbium trifluoride, mixed oxides and copolymers (80–81 wt%) Tetric N-Flow, Ivoclar: Ba–Al–silicate glass, copolymer, mixed oxides, ytterbium trifluoride, silicone dioxide (40 wt%) G-aenial Universal Injectable, GC: silica, barium glass, Ultra Fine (150 nm) (69 wt% Aligner Connect, GC: N.D.	SBS	All acceptable
[26]	Enamel Hri, Micerium: Tricyclodecane dimethanol dimethacrylate, UDMA Bracepaste, American Orthodontics: methacrylic acid ester, activator, ethoxylated Bisphenol A, Dimethacrylate, Tetramethylene Dimethacrylate, Diphenyl (2,4,6-trimethylbenzoyl) phosphine oxide	Enamel Hri, Micerium: (0.005 μm–0.05 μm silicon dioxide fillers), (0.2–3.0 μm glass fillers) (74 wt%) Bracepaste, American Orthodontics: N.D.	Shape	Similar fidelity

BIS-GMA: Bisphenol A Diglycidyl Ether Dimethacrylate; UDMA: Diurethane Dimethacrylate; TEGDMA: Triethylene Glycol Dimethacrylate; BIS-EMA: Bisphenol A Polyethylene Glycol Diether Dimethacrylate; DCP: tricyclodecane–dimethanol dimethacrylate; EBPADMA: ethoxylated bisphenol A dimethacrylate; D3MA: ZrO_2_: zirconium dioxide; SiO_2_: silicon dioxide; N.D.: not disclosed.

**Table 2:** The characteristics of the studies in humans included in this systematic review.

Study	Tested Composite Resin (Chemical Composition)	Sample Size (*n*)	Follow-Up Period	Fillers	Evaluated Parameters	Criteria Evaluation	Conclusions
[27]	Z350 XT, 3M: Bis-GMA, UDMA, Bis-EMA Amelogen Plus, Ultradent: Bis-GMA	Not reported	Not reported	Z350 XT universal, 3M: silica, zirconia nanoparticles (20 μm) (72.5 wt%) Amelogen Plus, Ultradent: 76 wt%	Wear	Clinical comparison of surface degradation of attachments over time; method not standardized/reported	Amelogen: higher wear
[28]	G-aenial Universal Injectable, GC: Bis EMA, UDMA Tetric EvoFlow, Ivoclar: Bis GMA, UDMA, D3MA GC Aligner Connect, GC: Tetric Prime, Ivoclar: Bis GMA, UDMA, Bis-EMA	Not reported	Not reported	G-aenial Universal Injectable, GC: silica, barium glass, Ultra Fine (150 nm) (69 wt%) Tetric EvoFlow, Ivoclar: Ba–Al–silicate glass, copolymer, mixed oxides, ytterbium trifluoride, silicone dioxide (40 wt%) Tetric Prime, Ivoclar: Ba–Al–silicate glass, copolymer, mixed oxides, ytterbium trifluoride	Color	G-aenial: silica, barium glass (69 wt%); Tetric EvoFlow: Ba–Al–silicate glass + fillers (40 wt%)	G-aenial: best
[29]	Not specified	Not reported	Not reported	Not specified	Wear/failure	Not specified	More failures
[30]	Transbond XT, 3M: Bis-GMA, TEGDMA Tetric EvoCeram, Ivoclar: dimethacrylates	Not reported	Not reported	Transbond XT, 3M: silica, quartz (70 wt%) Tetric Evoceram, Ivoclar: barium glass, ytterbium trifluoride, mixed oxides and copolymers (82 wt%)	Esthetics	Transbond XT: silica, quartz (70 wt%); Tetric EvoCeram: barium glass, ytterbium trifluoride (82 wt%)	Transbond: worse

BIS-GMA: Bisphenol A Diglycidyl Ether Dimethacrylate; UDMA: Diurethane Dimethacrylate; TEGDMA: Triethylene Glycol Dimethacrylate; BIS-EMA: Bisphenol A Polyethylene Glycol Diether Dimethacrylate; D3MA: decanedioldimethacrylate.

**Table 3:** Comparison of performances of composite resins according to evaluated outcomes.

Outcome	Best-Performing Materials	Lower-Performing Materials	Main Findings/Trends
Shear Bond Strength (SBS)	Composite resins with high filler loads and increased viscosity, e.g., Filtek Z350 XT, 3M: Bis-GMA, UDMA, Bis-EMA, silica, zirconia nanoparticles (20 μm) (72.5 wt%)	Filtek Z350 XT Flowable Restorative, 3M: Bis-GMA, UDMA, TEGDMA, Bis-EMA zirconia/silica (65 wt%) Aligner Connect, GC: Octahydro-4,7-methano-1H-(indenediyl) bis(methylene) bismethacrylate,1,3,5-triazine-2,4,6-triamine, polymer with formaldehyde, 2,2′-ethylene- dioxydiethyl dimethacrylate, 2-(H-benzotriazol-2-yl)-p-cresol, UDMA. Inorganic filler content not disclosed.	All materials showed clinically acceptable SBSs (≈6–14 MPa). Higher filler content was frequently associated with improved mechanical stability; however, SBS values should be interpreted in context of complete bonding protocol.
Wear Resistance	Composite resins with high filler loads and increased viscosity, e.g., Filtek P60: Bis-GMA, UDMA, Bis-EMA, 0.01–3.5 μm zirconia/silica (83 wt%)	Flow Tain, Reliace: Bis-GMA, TEGDMA, silica, barium glass, fumed silica (60 wt%)	Higher inorganic filler reduces volumetric loss and improves long-term stability.
Surface Roughness	Composite resins with high filler loads and increased viscosity: 3M: Bis-GMA, UDMA, Bis-EMA, silica, zirconia nanoparticles (20 μm) (72.5 wt%)	Flowable composite resins (e.g., Aligner Connect under high irradiance) Aligner Connect, GC: Octahydro-4,7-methano-1H-(indenediyl) bis(methylene) bismethacrylate,1,3,5-triazine-2,4,6-triamine, polymer with formaldehyde, 2,2′-ethylene- dioxydiethyl dimethacrylate, 2-(H-benzotriazol-2-yl)-p-cresol, UDMA. Inorganic filler content not disclosed.	Higher-viscosity materials show smoother surfaces; flowable composite resins may increase roughness.
Microhardness (VHN)	Optimized flowables (e.g., Clearfil Majesty Flow) and composite resins with high inorganic filler contents and increased viscosity. Clear fill Majesty Flow: TEGDMA, hydrophobic aromatic dimeth- Acrylate, dl-Camphorquinone · accelera- tors ·	Variable depending on composition	Depends on filler type and polymerization; no consistent superiority.
Color Stability	Nanohybrid/high-filler composite resins (e.g., Omnichroma, G-ænial Universal Injectable)	Flowable composite resins, e.g., Tetric PowerFlow: Bis-GMA, Bis-EMA, UDMA, DCP, barium aluminum silicate glass, iso-filler copolymer mix, ytterbium fluoride (71 wt%)	Coffee and wine cause highest discoloration; higher filler improves stability.
Accuracy of Attachment Reproduction	High-viscosity composite resins with PET-G molds	Flowable composite resins	High-viscosity resins show better shape fidelity; flowable composites may cause excess.
Handling/Adaptation	Flowable composite resins. G-aenial Universal Injectable, GC: Bis EMA, UDMA, silica, barium glass, Ultra Fine (150 nm) (69 wt%)	Conventional nanohybrid composite resins	Flowable composite resins provide better adaptation and easier handling.
Overall Clinical Recommendation	Posterior: nanohybrid/high-viscosity composite resins	Anterior: selected flowable composite resins with high filler loads	Material choice should balance mechanical load and esthetic needs.

**Table 4:** Risk-of-bias analysis according to RoB 2 tool (Cochrane Library).

Authors (Year)	D1	D2	D3	D4	D5
[14]	Low	Moderate	Low	Low	Low
[16]	Low	Low	Low	Low	Low
[19]	Low	Moderate	Low	Moderate	Low
[20]	Low	Low	Low	Low	Low
[21]	Low	Low	Low	Low	Low
[22]	Moderate	Low	Low	Low	Low
[23]	Low	Low	Low	Low	Low
[24]	Low	Low	Low	Low	Low
[25]	Low	Low	Low	Low	Low
[26]	Moderate	Low	Low	Low	Low
[27]	Low	Low	Low	Low	Low
[28]	Low	Low	Low	Low	Low
[29]	Low	Moderate	Moderate	Low	Low
[30]	Low	Low	Low	Moderate	Low

D1: Bias arising from randomization process; D2: bias due to deviations from intended interventions; D3: bias due to missing outcome data; D4: bias in measurement of outcome; D5: bias in selection of reported result. Judgment: high, moderate, and low.

## Data Availability

Not applicable.

## Abbreviations

The following abbreviations are used in this manuscript:

3D	Three-Dimensional
BPA	Bisphenol A
Bis-EMA	Bisphenol A Ethoxylated Dimethacrylate
Bis-GMA	Bisphenol A Glycidyl Methacrylate
CAD	Computer-Aided Design
CAM	Computer-Aided Manufacturing
CI	Confidence Interval
ΔE	Color Difference
ΔE00	CIEDE2000 Color Difference
GC	GC Corporation (Dental Materials Manufacturer)
LED	Light-Emitting Diode
PET-G	Glycol-Modified Polyethylene Terephthalate
PICO	Population, Intervention, Comparison, Outcome
PRISMA	Preferred Reporting Items for Systematic Reviews and Meta-Analyses
Ra	Surface Roughness Average
RMS	Root Mean Square
RoB	Risk of Bias
RoB 2.0	Revised Cochrane Risk-of-Bias Tool for Randomized Trials
SD	Standard Deviation
SEM	Scanning Electron Microscopy
SBS	Shear Bond Strength
TEGDMA	Triethylene Glycol Dimethacrylate
UDMA	Urethane Dimethacrylate
VHN	Vickers Hardness Number
